# Predictors of never testing for HIV among sexually active individuals aged 15–56 years in Rwanda

**DOI:** 10.1038/s41598-024-52652-w

**Published:** 2024-01-26

**Authors:** Hosee Niyompano, Emmanuel Biracyaza, François Hakizayezu, Jean Claude Niyoyita, Jerome Ndayisenga, Jared Omolo, Aline Umubyeyi

**Affiliations:** 1 Department of Biostatistics and Epidemiology, School of Public Health, University of Rwanda, Kigali, Rwanda; 2 Centre for Interdisciplinary Research in Rehabilitation of Greater Montreal (CRIR), Montreal, QC Canada; 3 School of Rehabilitation, Faculty of Medicine, Université de Montréal, Montreal, QC Canada; 4 African Research and Community Health Initiative (ARCH Initiative), Kigali, Rwanda; 5 Centers for Disease Control and Prevention (CDC), Field Epidemiology and Laboratory Training Program (FELTP), University of Rwanda, Kigali, Rwanda

**Keywords:** Risk factors, Health care, Diagnosis, Disease prevention, Health services, Public health

## Abstract

Human Immunodeficiency Virus (HIV) testing services are known as the primary step in preventing the spread of HIV. However, access to these crucial services varies across regions within continents due to disparities in healthcare infrastructure, resources, and awareness. Approximately one in every five people living with HIV (PLWH) encounters obstacles in accessing HIV testing, notably in Eastern and Southern Africa, where geographical, resource, awareness, and infrastructure limitations prevail. Consequently, HIV remains a significant public health concern in these regions, necessitating expanded testing efforts to combat the HIV/AIDS disaster. Despite these challenges, there is a lack of scientific evidence on the prevalence of HIV testing and its determining factors in Rwanda. This study determined the prevalence of never being tested for HIV and its associated factors among sexually active individuals aged 15–56 who participated in the Rwanda AIDS Indicators and HIV Incidence Survey (RAIHIS). This cross-sectional study enrolled 1846 participants. The variables were extracted from the RAIHIS dataset and statistically analyzed using STATA software version 13. Bivariate and multivariate logistic regression models were employed to identify predictors of never having undergone HIV testing, with a 95% confidence interval and a 5% statistical significance level applied. The prevalence of non-testing for HIV was 17.37%. Being aged 15–30 years (aOR 2.57, 95%CI 1.49–4.43, *p* < 0.001) and male (aOR 2.44, 95%CI 1.77–3.36, *p* < 0.001) was associated with an increase in the odds of never testing for HIV. Further, those from urban area were less likely than those living in rural areas to have never tested for HIV (aOR 0.31; 95% CI 0.38–0.67; *p* < 0.001). Participants who were not aware of HIV test facilitates were more likely to have never undergone HIV testing (aOR 1.75; 95% CI 1.25–2.47; *p* = 0.031) than their counterparts. While the prevalence of HIV non-testing remains modest, the significance of youth, male gender, lack of awareness, and rural residence as influential factors prompts a call for inventive strategies to tackle the reasons behind never having undergone HIV testing. Further exploration using mixed methodologies is advocated to better comprehend socio-cultural impacts and causation relating to these identified factors.

## Background

After 30 years of a complex Human Immunodeficiency Virus/Acquired Immunodeficiency Syndrome (HIV/AIDS) epidemic globally, it is estimated that 37 millions of people are infected with the virus worldwide, with 3.7 million of them being children^1,2^. Sub-Saharan Africa (SSA) has the highest HIV/AIDS prevalence, with an estimated 5.7 millions of people living with HIV and a lower prevalence of HIV testing^1,3^. In South Africa, HIV is primarily transmitted through heterosexual sex, and over 2 million of those infected are men aged 15 and older^4,5^. Previous studies established that the prevalence of HIV is higher in women than in men, however, the disparity in HIV testing remains an issue. Every year, approximately 400,000 children are infected with HIV, with 90% (300,000) being transmitted from mother to child^2^. Further, men are two to three times more likely than women to transmit HIV to men. This could also be due to HIV virus concentrations^1,6^. Moreover, HIV testing services are recognized as the key step in preventing the spread of HIV. However, access to these crucial services varies across regions within continents due to disparities in healthcare infrastructure, resources, and awareness^7^. Approximately one in every five people living with HIV (PLWH) encounters obstacles in accessing testing, notably in Eastern and Southern Africa, where geographical, resource, awareness, structural, legislative, and infrastructure limitations prevail^3,8,9^. This concern presents an increase of PLWH who are unaware because they have were not tested for HIV to determine their illness status and suggest them important interventions and access saving-treatments to increase their quality of life and reduce the risk of transmitting the virus to others^10,11^. Being tested for HIV should play a role in reducing stigma and discrimination associated with HIV/AIDS by promoting awareness, health education, and understanding. In United States, it is estimated that more than 168,000 were unaware of their HIV status and United Nations Programme on HIV/AIDS (UNAIDS) estimated at ½ of 35 million of PLWH who were never tested for HIV^10^.

Moreover, Rapid HIV testing is a relatively new invention that is revolutionizing the HIV screening process and allowing low-resource countries to test more people without the need for a lab or expensive equipment. Rapid testing is particularly useful for testing at community health screenings and in mobile clinics since it is less expensive and produces results more quickly^12,13^. Patients can receive their results twenty to thirty minutes after being tested with this new technology, as opposed to weeks with traditional testing methods. Rapid testing virtually eliminates this issue, allowing more people to learn about their HIV status right away^12,14^. Theoretically, quick testing should have a significantly favorable influence on public health preventive initiatives to lower and finally eradicate HIV/AIDS transmission by making affordable testing available to more people and by raising the possibility that people will actually obtain their results. Rapid HIV testing does not, however, come without certain disadvantages. Rapid testing has certain severe flaws that are brought to light by testing errors and ethical issues^12,15^. Although these services are prominent, previous studies continue indicating a low access to HIV testing, which contrasts sharply with the population's claimed desire for testing or retested for HIV^16^. Despite substantial endeavors to involve community members in accessing HIV testing and becoming aware of their HIV status, the global uptake of HIV testing has not reached an optimal level. The insufficient awareness regarding HIV represents a bottleneck in commencing ART, impeding the achievement of sustainable development goals of eradicating HIV by 2030^17^. Some of the barriers to HIV testing include lack of awareness and knowledge, geographical challenges and inadequate healthcare infrastructure, socio-cultural barriers, financial constraints, lack of confidentiality, stigma and discrimination, and fear of a positive diagnosis^17–19^.

Numerous factors contributing to a reduced likelihood of HIV testing have been identified, encompassing socio-economic indicators and behavioral elements. Socio-economic outcomes, including younger age, male gender, lower educational attainment, inadequate income or poverty, unemployment, and job insecurity, have been associated with lower rates of HIV testing^20–22^. Behavioral factors, such as engaging in multiple sexual partnerships and substance use, also contribute to the reduced likelihood of undergoing HIV testing. Masculine norms play a role in men's participation in the HIV care continuum, influencing aspects like clinic attendance, ART initiation, and treatment continuation^3,23^. Notably, women tend to access HIV testing more frequently than men, indicating a health disparity^24^. Psychological influences, such as anxiety, fear of test results, stigma, depression, prejudice, and low risk perception, have been documented as additional barriers to accessing HIV testing, often resulting in late-stage diagnoses and delays in care coordination^25,26^. The integration of HIV testing into antenatal care (ANC) facilities has led to greater test coverage for women, with approximately 60.7% obtaining HIV testing as part of ANC^27^. Delays in HIV testing are attributed to factors like insufficient testing coverage and variations in testing motivations between men and women^12,28^.

A nationwide household survey in Brazil indicated that a substantial proportion of men (72.7%) and women (55.3%) had never undergone an HIV test, a trend observed in several African countries, including Zambia, Kenya, and Uganda^29–31^. Motivations for testing varied, with 53% of women attributing it to routine prenatal care and 53% of men voluntarily seeking testing^12^. In Canada, HIV testing patterns differed among various demographics, with higher rates of client-initiated testing observed among men who have sex with men (MSM), low-income populations, and residents of large cities. Women who were widowed, divorced, separated, or single were more likely to seek testing voluntarily^3,23^. Multi-level studies have established a link between individual and contextual factors in HIV testing. Previous research in Los Angeles and the USA found that individuals from areas with a high perceived risk had higher testing rates, irrespective of the reasons for testing, as influenced by contextual-level factors. Areas with a high perception of risk also had a higher prevalence of respondents reporting inconsistent condom use and having multiple sexual partners^12,32^. Further, practitioners, healthcare professionals, and public health authorities remain actively engaged in seeking novel and effective strategies to bring an end to the HIV/AIDS pandemic. This ongoing commitment involves exploring innovative approaches, advancing research, implementing preventive measures, expanding access to testing and treatment, addressing socio-economic barriers, combating stigma, and continually refining public health interventions. The collaborative efforts of these dedicated individuals and organizations aim to achieve the ultimate goal of eradicating HIV/AIDS and ensuring better health outcomes for communities worldwide^12,33^.

The country grapples with HIV/AIDS as a pressing public health concern, reporting a prevalence of 3% among individuals aged 15–64 years and 2.6% among those aged 15–49 years^34,35^. Despite significant strides in combating this issue through multifaceted interventions like immediate linkage to ART, condom availability, targeted testing, and education campaigns, HIV/AIDS remains a persistent challenge^36,37^. Outstandingly, the Rwanda AIDS Indicator and HIV Incidence Survey (RAIHIS) documented reasons for non-testing but did not delve into the factors linked to this group. While Rwanda has introduced measures such as HIV self-testing and annual prevention events to encourage testing, HIV continues to pose significant health risks, with a 0.27% incidence rate and substantial percentages of men (24%) and women (16%) who have never undergone testing for reasons yet unidentified^38,39^. To our knowledge, the prevalence and determinants of individuals never tested for HIV within Rwanda are yet to be explored, highlighting a critical research gap. So, this study aims to fill this scarcity by determining the prevalence and factors associated with individuals aged 15–56 years who have never undergone HIV testing, aligning with the UNAIDS target. By conducting this study, our results will contribute to inform targeted interventions and policies to bolster testing rates and combat the pervasive impact of HIV/AIDS in the country.

## Methods

### Study design and area

The "Rwanda AIDS Indicator and HIV Incidence Survey" (RAIHIS) represents a comprehensive research endeavor within Rwanda, aiming to evaluate diverse dimensions of HIV prevalence, incidence, and correlated factors. As a nationally representative survey, its primary goal is to offer insightful perspectives into Rwanda's HIV landscape, playing a crucial role in guiding public health policies and interventions^35^. Through rigorous data collection methods, the survey gathers information on HIV testing, knowledge, attitudes, and behaviors, encompassing aspects such as condom usage, multiple sexual partners, and awareness of preventive measures. It also captures demographic variables like age, gender, education, and residence^36^. Ultimately, previous studies have established that RAIHIS serves as a crucial tool in shaping evidence-based strategies to address the HIV epidemic, improve awareness, and enhance the well-being of individuals and communities in Rwanda^35^.

The RAIHIS had two parts, the first of which was a baseline survey and the second of which was a follow-up survey conducted a year later. The first sample stage's sample frame was the same as that of the 2010 Rwanda Demographic and Health Survey, which was employed during the sampling for the RAIHIS survey. The questionnaires were created for the second sampling stage (households and individual questionnaires). The RAIHIS involved individual interviews and the collecting of blood samples. This cross-sectional study used secondary data from the most recent RAIHIS conducted in 2013–2014. The sample of the RAIHIS database was composed of men and women from all 30 districts and 5 provinces, ranging age of 15–59 years. A total of 15.58% of women and 20.20% of men who were each individually questioned stated that they had never undergone an HIV test. By analyzing the collected data, researchers and policymakers gain a deeper understanding of the prevalence of HIV, associated risk factors, and disparities across different population groups. This knowledge aids in designing targeted interventions, prevention strategies, and education campaigns to effectively combat HIV transmission and reduce its impact on public health.

### Study population

The population targeted in the RAIHIS 2013–2014 was 13,816 participants from 6918 households while the sample of 14,222 respondents from 6792 households participated in the survey. The women were 7419 aged 15–49 while the male was 6803 aged 15–59 years old. During the data collection of RAIHIS survey, the respondent’s rate was 98.2% and 98.4% from the households and individual’s response respectively. The current study was focused on those who were sexual active from RAIHIS 2013–2014 survey where a total number of 1846 people sexual active were extracted in the RAIHIS 2013–2014 database where its population was located in all five provinces of Rwanda and in all 30 districts. Per each district, 16 villages were selected, except in Kigali City and as a result 20 villages were selected included in the survey. Per each selected village, 14 households were designated, apart from the selected villages of Kigali City where 14.5 roughly to 15 households were selected^35^.

### Procedures and data collection

The RAIHIS questionnaire was divided into the households-based questionnaire and the individual-based questionnaire. This RAIHIS survey focused on the individuals who were sexually active. In this data collection, 15 households were selected from 20 selected Villages in Kigali city, while 14 households were selected from 16 Villages selected in the four provinces that remained excluding Kigali city. We extracted the variables of interest from the RAIHIS 2013–2014 dataset^35^. We had two types of variables namely dependent variable and explanatory variables. Firstly, the dependent variable of this study was self-reported lifetime history of HIV testing among participants. In the questionnaire, we asked respondents whether they have ever tested for HIV (‘have you ever tested for HIV?’). From this question, a binary outcome variable was produced, and coded as “1” never tested for HIV and “0” ever tested for HIV (yes = 1, no = 0). Secondly, the present study selected explanatory variables of never tested for HIV based on literature review^40–43^ and the range of RAIHIS 2013–2014 dataset^35^. The chosen independent variables are three categories; (a) socio-demographic characteristics that included age group, sex, province, type of residence area, education level, and working in a public institution, (b) HIV knowledge, access to high-quality medical care, and behavioral influences which included condom reused after washing, condom protects against sexually transmitted disease, condom contains HIV, and buying a condom, knowing where a person can buy a condom, awareness of HIV testing health facilities; and (c) HIV-related behaviors which included factors such as abstaining from having sex, limiting the number of sexual partners, avoiding sexual with sex workers, using a condom with a casual sex partner, using a condom at the last sex, knowing if the person with the last sex was HIV positive, number of sexual partners, and having sex with a non-married person.

### Data analysis

Descriptive statistics, including frequencies and percentages, were calculated to characterize the study population in terms of socio-demographic and pertinent variables. Variables showing a significance level of p < 0.25 in bivariate analysis were subsequently included in multivariate analysis. Multiple logistic regression models were employed to assess the presence of associations between independent and dependent variables. A statistical significance threshold of p ≤ 0.05, along with a 95% confidence interval, was utilized as the criterion to identify statistically significant associations between the independent and dependent variables. The analyses were performed using Stata 13.0 software (StataCorp, USA)^44^. We also used this software to evaluate the appropriateness of the logistic regression models used in this study and to detect multicollinearity using the Variance Inflation Factor (VIF), which measures how much the variance of a regression coefficient is inflated due to multicollinearity in the model. Due to this assessment, we removed from the model all the variables with VIF > 3.

### Ethics

This study was reviewed and approved by the ethics committee of the Rwanda Ministry of Health (MoH) through the Rwanda Biomedical Centre (RBC) that authorized the authors to access the RAIHIS 2013–2014 dataset from HIV division. Although the authors were provided authorization, all data were previously collected in accordance with the regulations and principles of the Helsinki Declaration^45^, since the data collection was approved by that Rwanda National Ethics Committee. Prior to this research, participants over the age of 18 provided informed consent. Those under the age of 18 signed assent forms, while their guardians signed consent forms to provide their data. All data were collected anonymously, and no geographical information was provided.

## Results

### Socio-demographic characteristics and prevalence of not being tested for HIV

Among the 1846 subjects, 296 individuals (17.39%) did not undergo HIV testing. The majority of these individuals were aged 15–30 years (n = 1583, 85.8%), identified as male (n = 1062, 57.53%), and were residents of Kigali city (n = 494, 26.76%). Over half of the participants (n = 1060, 61.92%) had received primary education, and the majority hailed from rural areas (n = 1230, 66.63%) (Table 1).

**Table 1:** Socio-demographic characteristics and prevalence of not being tested for HIV (n = 1846).

Variables	Frequency	Percent
HIV testing		
Ever tested	1525	82.61
Never tested	321	17.39
Age group		
31–56 years	263	14.2
15–30 years	1583	85.8
Gender		
Female	784	42.47
Male	1062	57.53
Province		
City of Kigali	494	26.76
East	399	21.61
North	218	11.81
South	415	22.48
West	320	17.33
Residence		
Rural	1230	66.63
Urban	616	33.37
Education level		
University	145	7.85
Advanced secondary education	351	19.01
Ordinary level	237	12.85
Primary education	1113	60.29
Working in public institution		
Yes	1082	58.61
No	764	41.39

### Knowledge, access to high quality of care and HIV-risk behaviors

The findings from this study provide valuable insights into participants’ attitudes, behaviors, and awareness related to sexual health, helping to shape targeted interventions and public health strategies. Moreover, majority (n = 1802, 97.62%) of participants showed that they were aware of where to get HIV testing. Remarkably, the results indicated that a majority (n = 1455, 78.82%) had not multiple sex partners. Further, 71.08% of participants (n = 1251) reported having an unlimited number of sexual partners. In the study participants, 45.61% (n = 842) did sexual intercourse with non-legal married partners. Furthermore, Encouragingly, 67.52% (n = 1189) chose not to partake in sexual activity (Table 2).

**Table 2:** Knowledge, Access to high quality of care and HIV-risk behaviors (n = 1846).

Variables	Frequency	Percent
Condom use at last sex		
Yes	795	43.04
No	1051	56.96
Multiple sex partner (> 1 partner)		
No	1455	78.82
Yes	391	21.18
Sex with legal non-married person		
No	1004	54.39
Yes	842	45.61
Awareness of HIV testing health facilities		
Yes	1802	97.62
No	44	2.38
Avoid sexual with sex workers		
Yes	498	26.98
No	1348	73.02
Abstain from having sex		
Yes	1189	64.41
No	657	35.59

### Socio-demographic, health-related and behavioral characteristics of the sexual active people

Our results showed prevalence of non-tested for HIV by socio-demographic characteristics. Those aged 15–30 years were almost twice to experience non-tested for HIV when compared to their counterparts and significantly presented a higher prevalence (n = 270, 91.21%) than those aged 31–56 years. Gender was significantly associated with non-tested for HIV with a higher prevalence in males than the females (n = 204, 68.92%). Regarding the residence, those from rural areas had a higher prevalence of non-tested for HIV (n = 184, 62.16%) than those from suburb and urban settings. In addition to that, education was significantly associated with non-tested for HIV (Table 3).

**Table 3:** Socio-demographic characteristics by the non-tested HIV status (N = 296).

Variables	Never tested for HIV		Statistical tests	
	Number	Percent	cOR (95% CI)	p-value
Age group (years)				
31–56 years	26	8.78	1	
15–30 years	270	91.21	1.90 (1.24–2.92)	0.003*
Gender				
Female	92	31.08	1	
Male	204	68.92	1.78 (1.36–2.33)	< 0.001**
Province Residence				
City of Kigali	91	30.7	1.40 (0.88–2.20)	0.147
East	70	23.6	1.31 (0.82–2.11)	0.250
North	29	9.8	0.78 (0.61–1.23)	0.446
South	57	19.3	1.17 (0.72–1.91)	0.506
West	49	16.6	1.13 (0.69–1.87)	0.612
Type of residence area				
Rural	184	62.16	1	
Urban	112	37.84	0.49 (0.32–1.76)	< 0.001**
Education level				
University	19	6.42		
Advanced secondary	25	8.44	1.09 (0.54–2.21)	0.802
Ordinary level	44	14.87	0.82 (0.45–1.49)	0.515
Primary school	208	70.27	1.99 (1.22–3.26)	0.006*
Working in public institution				
Yes	102	34.46	1	
No	196	65.54	5.04(1.82–6.39)	0.022*

*Statistical significance at p < 0.05, **statistical significant at p < 0.01.

### HIV-related knowledge, behaviors and access to healthcare services

Out of 296 individuals with never being tested for HIV, a majority (n = 183, 61.82%) was abstained from having sex. Regarding limit number sexual partners, a majority (n = 211, 71.28%) was not limited to the number of sexual partners. More than three quarters (n = 235, 79.4%) did not avoid sexual behaviors with sex workers. Further, a majority of non-tested people for HIV (n = 186, 62.84%) used condoms with casual sex partners. the findings also indicated significant association between utilization of condoms with casual sex partners (cOR = 1.38; 95%CI: 1.05–1.82, p = 0.021). More than a half of non-tested people for HIV (n = 171, 57.77%) did not use condoms at their last sexual intercourse. While there was a significant association between having multiple sexual partners, only 15.54% of participants had multiple sexual partners and 43.92% of participants did sex with non-married partner (Table 4).

**Table 4:** Knowledge on HIV and access to high quality of medical care from study participants.

Variables	Never having tested for HIV		95% for confidence internals	
	N	%	cOR (95% CI)	p-value
Abstain from having sex				
Yes	183	61.82	1	
No	113	38.18	1.12 (0.85–1.47)	0.399
Awareness of HIV testing health facilities				
Yes	274	92.57	1	
No	22	7.43	2.36 (1.1–4.08)	< 0.001**
Avoid sexual intercourse with sex workers				
No	211	71.28	1	
Yes	85	22.97	1.31 (0.97–1.77)	0.070
Multiple sexual partners (> 1 partner)				
No	250	84.46	1	0.122
Yes	46	15.54	0.87 (0.73–1.03)	
Sex with non-married partner				
No	166	56.08	1	
Yes	130	43.92	0.87 (0.73–1.03)	0.123

*NA* Not applicable, *HIV*: human immunodeficiency virus.

*Statistical significance at p < 0.05, **statistical significant at p < 0.01.

### Multivariate logistic regression models for the predictors of non-tested for HIV

Multivariate logistic models analyses showed relationships between age and non-tested for HIV with younger individuals having greater intention for not testing for HIV compared with older individuals (aOR 2.17; 95% CI 1.49–4.43; *p* < 0.001). Males were more likely to not intending to test for HIV (aOR 2.44; 95% CI 1.77–3.36; *p* < 0.001) when compared to females. Those who did not used the condoms during the intercourse were more likely to experience non-HIV testing (AOR = 1.44; 95% CI: 1.06–1.96; *p* = 0.019) than their counterparts. Low awareness of HIV testing services was associated with an increase of odds to be non-tested HIV (aOR = 1.75; 95% CI: 1.25–2.47; *p* = 0.031). Further, those who worked in a public institution were almost twice more likely to experience non-tested for HIV (aOR = 1.69; 95% CI: 1.26–2.019; *p* < 0.001) than their counterparts. Individuals with multiple sexual partners were almost twice more likely to experience non-tested for HIV (aOR = 1.66; 95% CI: 1.09–2.52; *p* = 0.017) compared to those who had one partner. Individuals from urban were less likely to have not been tested for HIV (aOR = 0.31; 95% CI: 0.38–0.67; *p* < 0.001) when compared to those from rural areas (Table 5).

**Table 5:** Multivariate logistic regression for the predictors of not being tested for HIV.

Variables	aOR; 95% confidence internals	p-values
Age group		
31–56 years	1	
15–30 years	2.57 (1.49–4.43)	< 0.001**
Gender		
Female	1	
Male	2.44 (1.77–3.36)	< 0.001**
Type of residence		
Rural	1	
Urban	0.31 (0.38–0.67)	< 0.001**
Multiple sexual partners (> 1 partner)		
Yes	1	
No	1.66 (1.09–2.52)	0.017*
Awareness of HIV testing health facilities		
Yes	1	
No	1.75 (1.25–2.47)	0.031*
Working in a public institution		
Yes	1	
No	1.69 (1.26–2.09)	< 0.001**
Educational level		
University	1	0.857
Advanced secondary	1.36 (0.67–2.75)	0.062
Ordinary level	1.25 (0.67–2.31)	0.071
Primary	1.17(0.92–1.39)	0.477

*NA* Not applicable, *aOR* Adjusted odd ratio, *HIV* human immunodeficiency virus.

*Statistical significance at p < 0.05, **statistical significant at p < 0.01.

## Discussion

The prevalence of individuals within the sexually active population who had not undergone an HIV test, as determined by RAIHIS, stood at 17.39% (296 out of 1702). Notably, a majority of these individuals were male, constituting 20.75% of the total. This prevalence markedly contrasts with the findings from Soweto, South Africa, as reported by Sakhile et al., where over two-thirds (71%) of men had not undertaken HIV testing^1,46,47^. Furthermore, our documented prevalence is also lower than that found in the Rwanda AIDS/HIV targets, where it was identified that 86.6% of Female Sex Workers (FSW) and 63% of Men who have Sex with Men (MSM) had undergone an HIV test within the 12 months preceding the survey^46^. This divergence in prevalence can be attributed to Rwanda's committed implementation of UNAIDS' 90–90–90 targets. These targets advocate that 90% of individuals with HIV are aware of their status, 90% of diagnosed individuals receive ART, and 90% of those on ART achieve viral suppression. As a result of this concerted effort, the prevalence of individuals who have not been tested for HIV has notably diminished^48^.

Our results revealed that young sexually active individuals were more likely to experience non-tested for HIV when compared to older people. The younger generation claimed that they should be more certain of their HIV-negative status than the older generation, but they had never had an HIV test and were reluctant to do so out of fear that others would find out. The results were supported by the other studies^1,49^. In spite of a heightened prevalence of HIV infection among women compared to men in the Eastern and Southern regions of Africa, women show greater rates of HIV testing, treatment initiation, and viral suppression than men. This disparity contributes to a reduction in AIDS-related mortality rates and their associated impacts, particularly benefiting women in contrast to men^22^. The findings from this study echo this evidence, indicating a higher likelihood of never having been tested for HIV among males. Nevertheless, these outcomes contrast with recent studies that have reported women being at a greater risk of never having undergone HIV testing^3,50^.

To concur with prior studies^51–53^, our findings revealed that participants whose age was 15–30 years had a higher likelihood of not undergoing HIV testing compared to those aged 31 and above. This trend could stem from the younger demographics’ apprehension about learning their HIV status. The reason should be that the young were feared to know their HIV status, they should be confident that they are HIV negative, and they should also be more afraid of others knowing their HIV status than the elderly. This was consistent with the findings of the Soweto study, which discovered that the majority of those who did not have an HIV test were young people^1,53^. Further, the findings indicated that males had higher rate of never being tested for HIV than females. This is relevant to the evidence from prior study conducted in Gambia and New Guinea^52,54^. Subsequently, our analysis revealed that males exhibited a greater likelihood of never having undergone an HIV test in comparison to females. The reason for this is that men may have more sex partners than women, which causes them to be concerned about their HIV status. This was consistent with RAIHIS findings, which found that 80.2% of men and 84.85% of females self-reported having ever had an HIV test, implying that 19.8% of men and 15.15% of females had never had an HIV test^35^. Thus, females engaging in riskier behaviors (such as multiple sexual partners) had a lower likelihood of late retesting, suggesting a realistic assessment of their potential risk of HIV infection.

The usage of condoms is also important for HIV testing; according to the study, people who do not use one with a casual sex partner were less likely to get their HIV tested than those who do. This implied that you are exposed to HIV once you engage in unprotected sex and are also afraid to seek an HIV test. Rwandan traditions, however, permit the majority to believe that purchasing condoms is shameful. In contrast to those who disagree, those who believe that this was not tested were more likely. This implied that if purchasing a condom makes you feel embarrassed, you should engage in unprotected sex rather than fearing finding out your HIV status and so lower the rate of HIV testing. However, the study discovered that those who are unaware of where one can go for an HIV test are more likely to not get tested in comparison to those who are aware^3,28^. The individuals whose residence was rural were at higher risk to have never tested for HIV which could be due to lower socio-economic outcomes when compared to those from urban settings^2,13,31^. In concur with the previous studies^9,16,18^, participants with low awareness of where to get HIV testing were almost twice more likely to never get HIV testing compared to their counterparts.

In alignment with previous research^31,55^, awareness of condom availability emerged as a significant protective factor against abstaining from HIV testing. This observation underscores the notion that a notable proportion of individuals opting against HIV testing are likely uninformed about condom purchasing locations and HIV testing facilities^1,56^. The cause may be that they avoid unprotected sexual activity out of concern that they will be HIV-tested if they don't know where to buy a condom. Men's knowledge of their HIV status is crucial for HIV prevention since it has been linked to a reduction in risky behavior among those who have tested positive for the virus^1,56^.

Our study indicated considerable associations between low levels of education and not getting an HIV test among people with elementary degrees as compared to those with secondary degrees. The reason may be that one of the obstacles to participation in the voluntary testing and testing cancellation among the participants is literacy. This was the same as what was discovered in Soweto, where men who were sexually active had lower levels of education and a high correlation between not testing for HIV^1,57^. There was strong associations between not getting tested for HIV and one's line of work that could be because people who work in the public sector are significantly more protected from getting tested than people who do not. Our results revealed that those who worked in private institutions were more likely to get tested for HIV than those from public institutions. This discrepancy suggests that there could be a specific condition for employment within the public sector, possibly mandating an HIV test as part of the employment criteria. Additionally, working in a public institution can potentially influence the decision to not get tested for HIV due to various factors like stigma, fear of confidentiality breaches, or lack of access to private healthcare. These results are relevant to the previous in Tanzania^58^.

Several limitations of this manuscript should be acknowledged. First, the data on the entire study population were collected at a single point in time to examine the relationship between outcome and dependent variables. As a result, a cross-section study provides a snapshot of the frequency of the outcome in the population at a given point in time. Everyone should read this study because it is difficult to tell whether the exposure or the outcome came first, and there may be reverse causality and direction (it is difficult to drive causal relationship and direction of the cross-section study). It was also difficult to determine whether the outcome came first or whether the exposure came first. Second, the current study encountered a study design limitation in that it was unable to draw causal inferences from the documented associations. Third, because our study is based on secondary data, it should be limited in its ability to address all relevant research questions and may not contain all of the details that the researcher would like to know. Furthermore, it is possible that the data was not collected with the specific demographic the researcher is interested in studying, during the desired years, or in the desired geographic area. Fourth, some important factors such as behavioral factors (smoking, sexual orientation, sexual orientation, marital status), psychological factors (such as anxiety, depression), and clinical factors (such as complicated disease diagnosis) were not investigated for their influences on HIV testing. This study had significant strengths that should be highlighted in addition to its shortcomings. First off, this is the first study to examine characteristics associated with non-tested HIV status using a large sample size. Second, methodological validity was a top priority in the design of this investigation. Multistage probability sampling was used to sample at the provincial level with detailed mapping, and if participants weren't at home, efforts were made to visit sampled households many times. Therefore, our results might be used as a reference point for next population-based studies on HIV testing in Rwanda. While we acknowledge that changes in socio-cultural context, policies, and interventions might have occurred, the fundamental insights gleaned from this dataset can still inform ongoing and future interventions aimed at promoting HIV testing.

## Conclusion

The prevalence of sexually active individuals aged 15–56 years who haven't had HIV testing remains a public health concern. Nonetheless, various significant factors contribute to this, including age, low awareness of where to get HIV test, residing in rural areas, educational level, being male, and working environment. To address this, efforts should aim at breaking barriers to testing, dealing with concerns about confidentiality and societal stigma linked to HIV testing among these socio-demographics. Boosting educational campaigns in rural regions to raise awareness about testing sites and preventive measures, like condom availability, could notably bolster testing rates. Moreover, making information accessible and launching community-based programs can bridge knowledge gaps, ensuring equitable access to testing regardless of educational levels. There is a need for tailored strategies to engage sexually active individuals and the wider populace effectively. In Rwanda, interventions should blend personalized risk approaches with culturally fitting HIV infection and testing information. Addressing these barriers through the recommended actions aligns with the broader objectives of Sustainable Development Goal (SDG 3), paving the way for improved health outcomes, reduced HIV/AIDS prevalence, and the overall well-being of communities in Rwanda. These interventions and strategies, if implemented effectively, can significantly contribute to the achievement of SDG 3 targets by 2030.

### Future directions

Based on our scientific evidences, more research efforts should investigate deeper into the behavioral and psychological aspects influencing HIV testing. Exploring factors like smoking, mental health conditions, and sexual orientation in relation to testing behaviors could offer comprehensive insights into the complexities surrounding testing reluctance. Further, longitudinal studies are crucial to discern causal relationships between identified predictors and non-tested HIV status. These studies could unveil the temporal sequence of factors leading to testing avoidance, helping in the development of targeted interventions and policies. Additionally, integrating qualitative methods alongside quantitative analysis could uncover nuanced reasons behind testing behaviours, capturing personal experiences, and socio-cultural influences. Understanding these intricacies is critical in tailoring interventions that resonate with diverse societal contexts. Lastly, ongoing monitoring and evaluation of HIV testing programs are essential to gauge the effectiveness of implemented interventions.

## Data Availability

The datasets used and/or analysed during this study are available from the corresponding author on reasonable request.
